# Cellular Metabolic Responses to Copper Nanoparticles: Comparison Between Normal and Breast Cancer Cells

**DOI:** 10.3390/ijms262110716

**Published:** 2025-11-04

**Authors:** Alexandra Ivan, Maria-Alexandra Pricop, Alexandra Teodora Lukinich-Gruia, Iustina-Mirabela Cristea, Adina Negrea, Ioan Bogdan Pascu, Crenguta Livia Calma, Andreea Paunescu, Virgil Paunescu, Calin Adrian Tatu

**Affiliations:** 1 Department of Functional Sciences, Center of Immuno-Physiology (CIFBIOTEH), University of Medicine and Pharmacy “Victor Babes”, Eftimie Murgu Sq. 2, 300041 Timisoara, Romania; ivan.alexandra@umft.ro (A.I.); crenguta.calma@umft.ro (C.L.C.); vpaunescu@umft.ro (V.P.); tatu.calin@umft.ro (C.A.T.); 2 OncoGen Centre, Clinical County Hospital “Pius Branzeu”, Blvd. Liviu Rebreanu 156, 300723 Timisoara, Romania; maria.pricop@student.upt.ro (M.-A.P.); miracristea82@gmail.com (I.-M.C.); 3 Department of Applied Chemistry and Environmental Engineering and Inorganic Compounds, Faculty of Industrial Chemistry, Biotechnology and Environmental Engineering, Politehnica University Timisoara, Vasile Parvan 6, 300223 Timisoara, Romania; adina.negrea@upt.ro; 4 Research Institute for Renewable Energies-ICER, Politehnica University of Timisoara, 138 Gavril Musicescu Street, 300774 Timisoara, Romania; ioan.pascup@upt.ro; 5 Faculty of General Medicine, “Carol Davila” University of Medicine and Pharmacy, Eroii Sanitari Bvd., No. 8, Sector 5, 020021 Bucharest, Romania; andreea.paunescu@stud.umfcd.ro

**Keywords:** nanomaterials, metabolomics, in vitro toxicity, oxidative stress

## Abstract

The use of copper nanoparticles (CuNPs) seems to be an alternative therapeutic strategy for cancer therapy due to low-cost synthesis and anticancer activity. In this work, CuNPs’ effects were tested in various concentrations on two types of cells: mesenchymal stem cells (MSCs) and a breast cancer cell line, SKBR3. The concentrations (0.25 mM, 0.5 mM, 1 mM and 2 mM) were first tested on an impedance-based cytotoxicity assay and then used in further cellular metabolic assays. Next, several techniques were applied to test the chosen concentrations: assessment of apoptosis, intracellular reactive oxygen species (ROS) levels, oxidative stress-related gene expression, assessment of mitochondrial respiration and fatty acid methyl ester (FAME) profile evaluation. The higher CuNP concentrations tested on the SKBR3 cell line showed a dose-dependent decrease in the cell index. SKBR3 cells displayed increased *CAT* and *SOD* expression, revealed by strong dose-dependent fluorescence. Annexin/PI staining confirmed increased SKBR3 cell death induced by the higher doses of CuNPs. SKBR3 revealed higher baseline respiratory capacity compared to MSCs. Fatty acid methyl esters (FAMEs) are in higher abundance in MSCs compared to the SKBR3 cell line. The different metabolic response in the tested cells to the CuNPs’ presence could help establish a future personalized treatment for breast cancer patients.

## 1. Introduction

Nanotechnology and cell therapy are novel approaches currently used in biomedical research as well as in therapy. The rapid developments in the field of nanotechnology offers novel horizons for medical applications, from drug delivery to disease detection [1]. At the same time, advancements in the field of nanoparticle (NP)-based treatments and diagnostics for cancer have opened new possibilities that require further investigations [2]. One group of the potential agents for cancer treatment are metal NPs, largely due to the abundance of their oxides forms in the environment, resulting in a cost-effective synthetic production approach [3]. Nanotechnology has become a main subject in medicine [2] through the introduction of nanomaterials with new physical, chemical and biological properties [4]. There is a considerable interest in metal nanoparticles due to their special properties and possibility for biomedical applications [2]. Metal nanoparticles (NPs), among all the NPs, serve as multipurpose agents [3]; they have a small size and large specific surface area. Copper is an essential trace element critically involved in various cellular processes [5]. Copper, when used in the form of NPs, represents a promising alternative in biomedicine due to its multifaceted properties ranging from antibacterial and antifungal activities to potential cost-effective cancer treatments [6]. However, for NPs to be safely applied in medicine, their synthesis must be optimized to limit adverse effects in healthy tissues, as NPs have the potential to induce oxidative stress, inflammation and cytotoxic effects [1]. CuNPs are obtained through various techniques, the most common one being chemical synthesis by chemical reduction [7,8] with copper-based precursors as reducing agents [9]. This method is selective and could control the nanoparticles’ final shape, size and surface properties, with these being important factors for their interaction with cells [8]. As the size of the material decreases to nanoscale level, its physical and chemical properties change significantly. These unique characteristics make NPs extremely valuable for biomedical applications; however, at the same time, careful consideration must be given in order to prevent unwanted side effects in healthy tissues [9]. The synthetically produced CuNPs need an in vitro toxicity evaluation to validate their medical applications. Therefore, cell models are utilized, and CuNPs are tested in assays like cell viability/toxicity, oxidative stress and ROS generation [10]. CuNPs also showed effects on different types of cancer cells leading to a novel or alternative method to chemotherapy [11]. For effective therapy, drugs must reach their targets with minimal off-target effects [1]. CuNPs’ cytotoxic effects arise from their accumulation, dissolution and electrostatic adhesion to cells. This process disrupts cellular membrane integrity, facilitates the entry of CuNPs and copper ions inside the cells, generates oxidative stress, leads to DNA damage and causes the inhibition of cell proliferation [12,13]. Copper nanoparticles’ cytotoxic effects on cancer cells translate into oxidative stress, DNA damage and inhibition of cell proliferation followed by cellular necrosis or apoptosis [12], with their use in cancer treatment being also described [3]. Copper induces oxidative stress, also causing metabolic dysregulation and alterations in lipogenic metabolism. These actions interfere with the expression and activities of antioxidant enzymes, such as superoxide dismutase (*SOD*) and catalase (*CAT*), inducing oxidative stress and increasing ROS generation [14]. Oxidative stress and increased ROS contents are the main cause for Cu-induced cytotoxicity [15]. Fatty acid (FA) metabolic pathways are important in cancer development, because of FAs’ roles in membrane formation, gene expression, cell functions and intracellular signaling [16]. To evaluate CuNPs’ effects on cellular models, metabolomics combined with other scientific techniques need to be applied to obtain a panel of possible biomarkers [10].

In this study, the biological properties of spherical copper nanoparticles (CuNPs), previously synthesized [8] through a two-step chemical reduction process with sodium borohydride, were assessed for the first time. The use of integrated comparative approaches adds novelty to our study by simultaneously evaluating normal and cancer cells under identical experimental conditions. Various CuNP concentrations were tested, and their effects were compared between normal mesenchymal stem cells (MSCs) versus SKBR3, a cancerous breast cell line. Therefore, MSCs were used as a normal cell model, while the SKBR3 line represented a suitable proxy for a breast cancer cell model. The tested concentrations were established through a cytotoxicity assay and further used to investigate their impact on additional cellular processes, including apoptosis, ROS generation, oxidative stress-related gene expression, mitochondrial respiration and fatty acid content. By directly comparing responses in normal versus cancerous cells, this work provides critical insights into the potential therapeutic applications and safety concerns of CuNPs.

## 2. Results

### 2.1. Scanning Electron Microscopy Analysis (SEM) and Energy-Dispersive X-Ray Spectroscopy (EDX)

**Figure 1:**
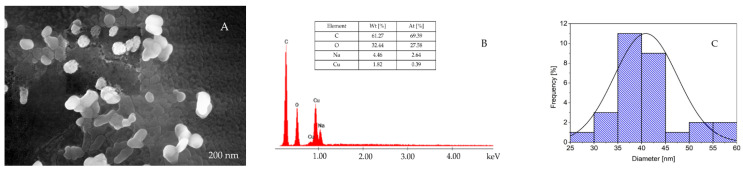
(**A**) SEM picture of CuNPs; (**B**) energy-dispersive X-ray spectrum (EDX) of the nanomaterial; (**C**) experimental determination of the mean diameter for CuNPs.

Copper nanoparticles (CuNPs) were synthesized via chemical reduction, and the optimal synthesis parameters were determined to be a molar ratio of Cu(II):TSC:BH_4_^+^ = 1:1:0.2, pH = 5, a homogenization time of 60 min and a temperature of 25 °C. By systematic variation of reagent ratios and reaction conditions, high concentrations and stable colloidal dispersions of spherical CuNPs were achieved, with their surface morphology being characterized by SEM (Figure 1A) and their elemental composition being quantified by EDX spectroscopy (Figure 1B).

**Figure 2:**
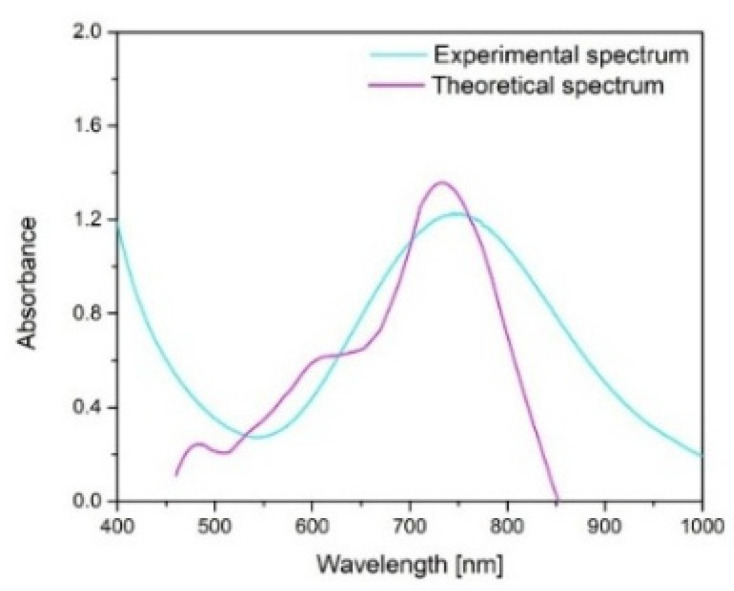
Theoretical evaluation of the CuNPs’ diameter.

The CuNPs’ morphological properties examined by SEM (Figure 1A) revealed that they form nanoclusters that are composed of uniformly dispersed spherical and quasi-spherical particles and that the presence of C, O and Na are specific to trisodium citrate (Figure 1B). In addition, in the EDX spectrum, the presence of Cu confirms the formation of copper particles. The carbon peak observed in the spectrum is attributed to the carbon tape used to mount the sample onto the stub. The average diameter of CuNPs was 37.5 nm (Figure 1C). To estimate a theoretical diameter for the spherical CuNPs, a spectrum using MiePlot software v.4.6. was created (Figure 2).

Based on the theoretical spectrum generated using MiePlot, the calculated diameter of CuNPs was approximately 30 nm, which is relatively similar to the 37.5 nm size measured by SEM. This highlights that the Mie theory provides a useful preliminary tool for determining the size of spherical or quasi-spherical CuNPs, while the broader full width at half maximum of the experimental spectrum and lower absorbance suggests a lower concentration and some polydispersity compared to the ideal monodisperse theoretical case.

### 2.2. Impedance-Based Cytotoxicity Assay

**Figure 3:**
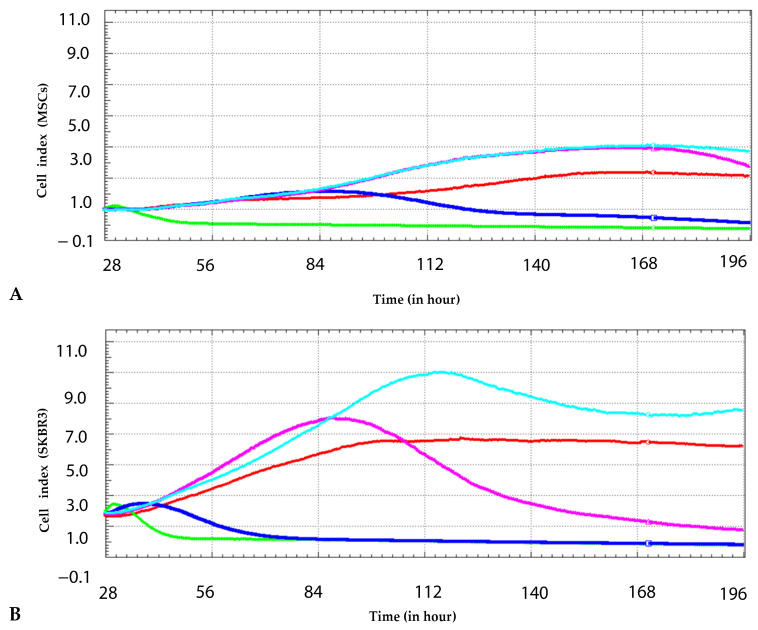
Real-time monitoring of cell index in MSCs (**A**) and SKBR3 cells (**B**) using the xCELLigence system. Cells were treated with CuNPs at different concentrations: 0.25 mM (light blue), 0.5 mM (magenta), 1 mM (dark blue) and 2 mM (green) for 7 days. Control is plotted in red.

To assess the cytotoxic effect of CuNPs on MSCs and SKBR3 cells, real-time cell monitoring was performed over seven days using the xCELLigence system. In MSCs (Figure 3A), the cell index remained relatively stable at lower concentrations compared to the control, showing minor deviation throughout the experiment and slightly exceeding the control cells, which suggests low-dose stimulation of cell growth. At the highest concentration of CuNPs (2 mM), a significant decline in the cell index was observed after 24 h. After 72 h post-treatment, a decline was also observed at the 1 mM concentration. In SKBR3 cells (Figure 3B), control cultures showed a steady increase in the cell index over time. At low concentrations (0.2–0.25 mM), the growth curves transiently surpass the control, indicating a similar response observed in MSCs. At 0.5 mM, a pronounced decrease was observed after 72 h, while at the highest concentration (1–2 mM), the cell index dropped sharply after 24 h, suggesting a severe cytotoxic effect and impaired proliferative capacity.

### 2.3. Assessment of Apoptosis via Annexin V FITC/PI Staining

**Figure 4:**
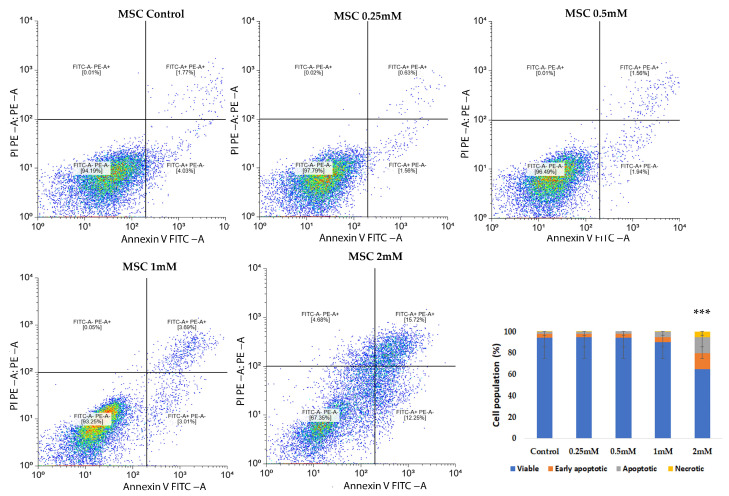
Annexin V and PI staining in MSCs treated with different concentrations of CuNPs. The lower left quadrant represents live cells (Annexin V, PI negative), the lower right quadrant represents early apoptotic cells (Annexin V positive, PI negative), the upper right quadrant represents late apoptotic cells (Annexin V and PI positive), and the necrotic or dead cells (Annexin V negative and PI positive) are presented in the upper left quadrants. The bar graph (mean ± SD from *n* = 3 independent experiments) summarizes the quantitative distribution of viable, apoptotic and necrotic cells across treatments, indicating a dose-dependent cytotoxic effect at higher CuNP concentrations. Statistical significance is denoted as follows: *** *p* < 0.001.

In MSCs, CuNPs induced a dose-dependent increase in the number of apoptotic cells, while the necrotic fraction remained low (4.6%), suggesting a predominantly controlled apoptotic response. A significant increase in apoptotic cell counts (*p* < 0.001) was observed mainly at the highest concentrations tested (2 mM). Representative dot plots illustrate the distribution of viable, early apoptotic, apoptotic and necrotic cells in response to CuNPs (Figure 4).

**Figure 5:**
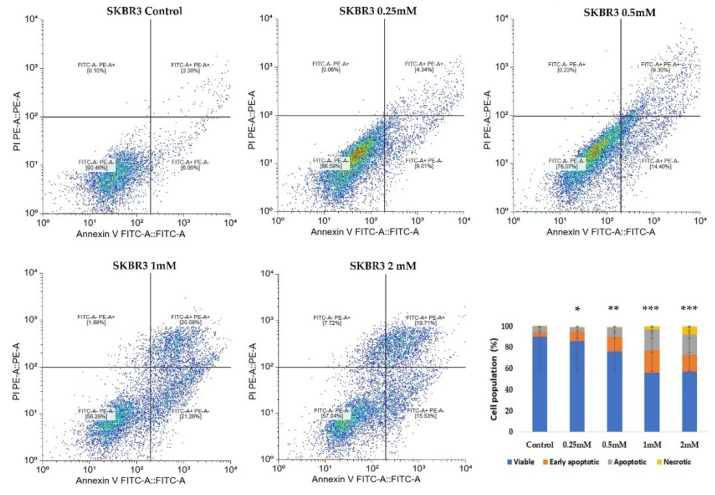
Annexin V and PI staining in SKBR3 cells treated with different concentrations of CuNPs. Representative dot plots illustrate the distribution of the viable, early apoptotic, late apoptotic and necrotic populations under each treatment conditions. The bar graph (mean ± SD from *n* = 3 independent experiments) summarizes the quantitative distribution of viable, apoptotic and necrotic SKBR3 cells following CuNP exposure, demonstrating a clear dose-dependent cytotoxic effect and increased sensitivity to higher CuNP concentrations compared to MSCs. Statistical significance is denoted as follows: * *p* < 0.05, ** *p* < 0.01 and *** *p* < 0.001.

In the SKBR3 cells, CuNP treatment for 24 h led to a similar redistribution across Annexin V/Pi quadrants. The proportion of apoptotic/necrotic cells increased in a dose-dependent manner, with the 2 mM concentration eliciting the strongest cytotoxic effect (*p* < 0.001), followed by the 1 mM concentration, which elicited much higher toxicity towards SKBR3 cells (20.5% Annexin V^+^ cells) compared to MSCs (3.68% Annexin V^+^ cells) (*p* < 0.001) (Figure 5). These findings indicate a higher sensitivity of SKBR3 cells to CuNP-induced cytotoxic effects compared to MSCs, which maintained a predominantly viable population even at higher doses.

### 2.4. Reactive Oxygen Species (ROS) Detection Assay

**Figure 6:**
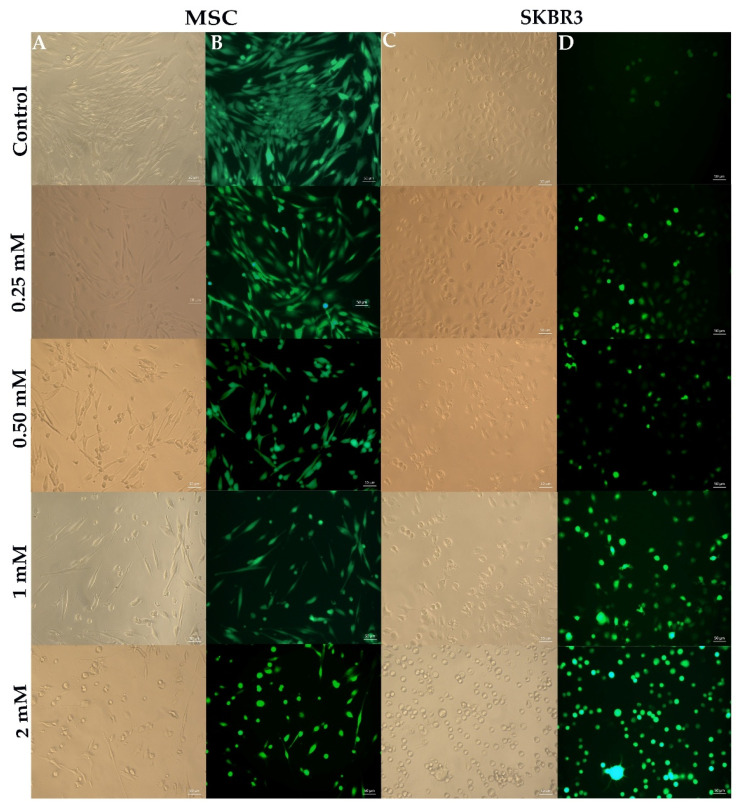
Intracellular ROS levels in MSCs and SKBR3 cells following CuNP treatments. Representative bright-field images for MSCs (**A**) and SKBR3 cells (**C**) and fluorescence images for MSCs and SKBR3 cells using the DCFH-DA fluorescence probe (**B**,**D**). Cells were exposed to increasing concentrations of CuNPs. Scale bar: 50 µm.

Fluorescence microscopy revealed distinct patterns of intracellular ROS levels, as observed following DCFH-DA fluorescent probe staining, between MSCs and SKBR3 cells after CuNP treatment (Figure 6). In MSCs, the control group displayed a distinctly higher fluorescence signal compared to SKBR3 controls, indicating a higher basal ROS level. In SKBR3 cells, the baseline fluorescence in controls was much weaker compared to MSCs. However, following CuNP treatment, SKBR3 cells exhibited increased fluorescence signal intensity with rising concentrations. Upon treatment, both MSCs and SKBR3 cells showed dose-dependent changes in cell morphology. At higher concentrations, the cells presented significantly altered morphology and reduced confluence, with numerous round-shaped cells detaching into suspension, indicative of stress response and cytotoxic effects.

### 2.5. Oxidative Stress-Related Gene Expression

**Figure 7:**
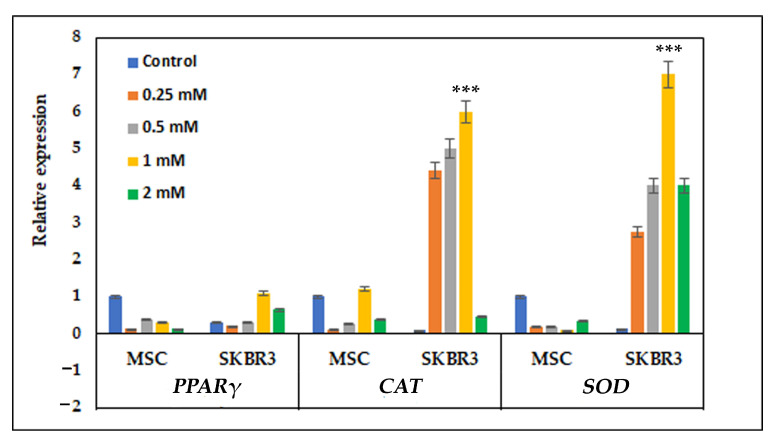
Antioxidant gene expression and cellular response to CuNPs. The expression of oxidative stress-related genes (*PPARγ*, *SOD* and *CAT*) was assessed by quantitative PCR, with *GAPDH* as the internal reference gene for normalization. Data are presented as the mean ± SEM from three independent experiments. Statistical significance is denoted as follows: *** *p* < 0.001. The oxidative stress-related genes (*PPARγ*, *SOD* and *CAT*) were represented via agarose gel electrophoresis (Figure S1).

Quantitative PCR reaction revealed distinct patterns of gene expression in MSCs and SKBR3 cells for all three genes analyzed (Figure 7). In MSCs, the relative expression of *PPARγ*, *CAT* and *SOD* decreased compared to the control. By contrast, the SKBR3 cells exhibited a clear and dose-dependent response. *PPARγ* expression remained relatively low compared to the control. *CAT* and *SOD* were strongly upregulated at higher CuNP concentrations, with maximal induction levels observed at 1 mM (*p* < 0.0001).

### 2.6. Assessment of Mitochondrial Respiration

**Figure 8:**
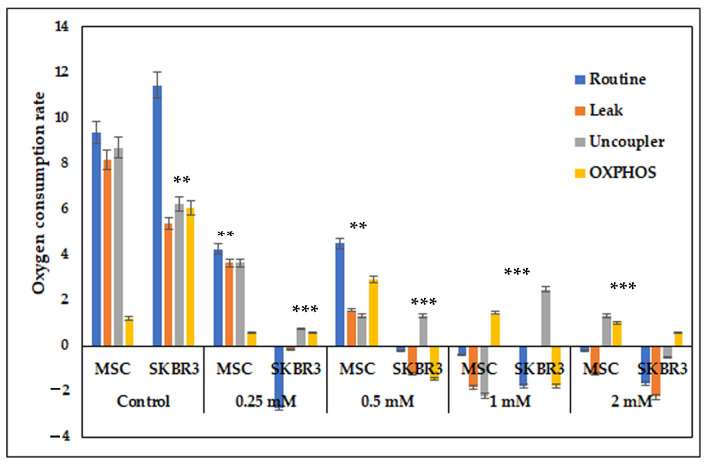
Oxygen consumption rates in MSCs and SKBR3 cells following CuNP treatments, assessed using the Oroboros 2k high-resolution spirometry system. The figure illustrates the effects of CuNPs on routine respiration (R), leak respiration (L), maximal respiratory capacity (M) and oxidative phosphorylation capacity (OXPHOS). Representative oxygraph depicting oxygen consumption dynamics and basal respiration are shown in the Supplementary Files (Figures S2 and S3). Statistical significance is denoted as follows: ** *p* < 0.01 and *** *p* < 0.001.

The evaluation of mitochondrial respiration indicated a progressive and dose-dependent disruption in response to CuNP treatment in both MSCs and SKBR3 cells (Figure 8). Under normal/control conditions, both MSCs and SKBR3 cells displayed active mitochondrial respiration, with SKBR3 exhibiting the highest values across routine and OXPHOS states, consistent with the elevated metabolic demand of tumor cells. MSCs showed balanced respiration with lower basal consumption, reflecting their slower metabolic turnover. Following CuNP treatment, both cell types showed sharp decreases in oxygen consumption rates compared to the controls. At higher concentrations (1–2 mM), mitochondrial respiration was completely inhibited in both MSCs and SKBR3 cells.

### 2.7. Assessment of Fatty Acid Content in MSCs and SKBR3 Cell Line

**Figure 9:**
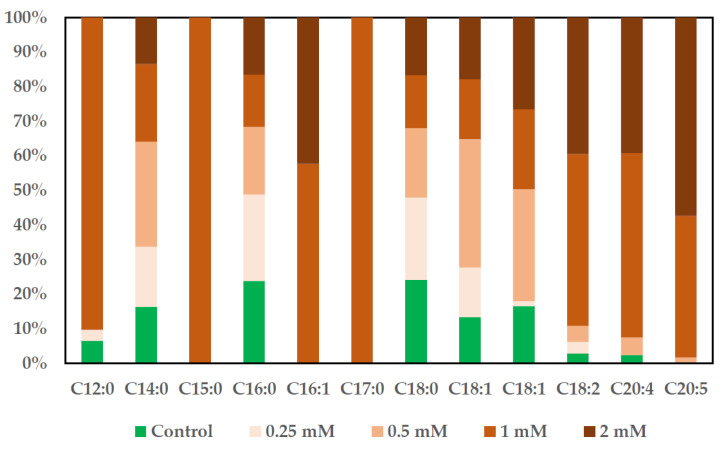
The effect of CuNPs on the proportion of FAMEs in the MSCs.

**Figure 10:**
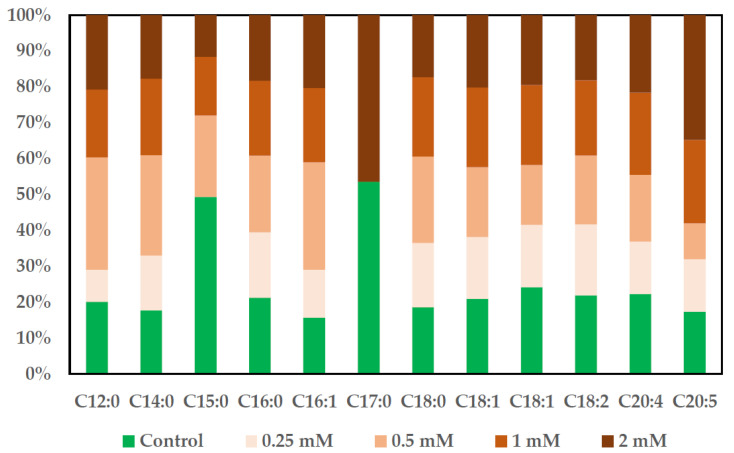
The effect of CuNPs on the proportion of FAMEs in the SKBR3 cell line.

The effect of CuNPs on the FAME profile in MSCs and the SKBR3 cell line was evaluated by GC-MS after Folch extraction [17], saponification and derivatization. These results are illustrated in representative chromatograms showing the methylated fatty acids (Figure S4). FAMEs determined in both cell lines were calculated and are represented in graphics, where the percentage of each individual FAME was calculated in each type of sample. The results obtained are represented in 100% stacked columns, where it can be seen how the percentage of each FAME contributes to the total number of fatty acids (Figure 9 and Figure 10).

MSCs (Figure 9) treated with CuNPs presented a different FAME profile compared to the SKBR3 cell line (Figure 10). The control and the 0.25 mM CuNP-treated samples had the same percentage in palmitic and stearic acids, followed by the other samples, with a difference of a 1.5 ratio. Regarding the other fatty acids from control samples, they were found to be at lower percentages than those from the MSCs treated with CuNPs. The results indicate that penta- and heptadecanoic acids were present only in MSCs treated with 1 mM CuNPs. Also, the 1mM concentration led to a higher percentage in lauric, palmitoleic, linoleic and arachidonic acids. Oleic and elaidic acids were found to be in higher percentages in the 0.5 mM treated cells, and the lower concentrations were found to be in the 0.25 mM treated cells. Regarding polyunsaturated (PUFA) FAMEs, these were found to be in higher proportions in the 1 mM and 2 mM treated samples. Arachidonic acid was missing from the 0.25 mM treated cells, while eicosapentaenoic acid was present only in the 0.5 mM, 1 mM and the 2 mM treated cells.

The general proportion of FAMEs in the SKBR3 cell line (Figure 10) was almost identical in the control and the CuNP-treated cells, albeit with some exceptions. For the SKBR3 cells, the percentage of heptadecanoic acid was found to be similar in the control and the 2 mM treated cells. Another peculiarity appears in the percentage profile of the pentadecanoic acid, which was found to be present in all samples except for the 0.25 mM treated cells. The lowest percentages of FAMEs were obtained in the 0.25 mM treated cells. The highest percentage of PUFA was obtained for the eicosapentaenoic acid in the 2 mM treated cells.

**Figure 11:**
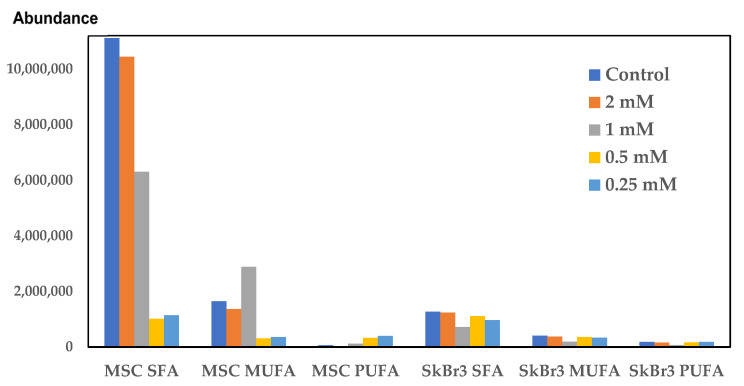
The effect of increasing CuNP concentrations on saturated (SFA), mono- (MUFA) and poly-unsaturated (PUFA) FAME levels in MSCs and SKBR3 cells.

Regarding the saturated and unsaturated FAME profiles, a difference between MSCs and SKBR3 cells could be seen. A common behavior of FAMEs is a decrease in all types of cells from the control to the lowest concentration (0.25 mM) of CuNPs (Figure 11). Although a slight decline in saturated fatty acid (SFA) levels is observed in both types of cells as CuNP concentration decreases, for the last two concentrations, the levels are similar. SKBR3 cells are richer in unsaturated fatty acids than MSCs, and overall, the 0.5 mM concentration is the one inducing a decrease in all types of fatty acids.

The low doses of 0.25 mM and 0.5 mM CuNPs have been found to promote an increase in both saturated and unsaturated fatty acids in both the MSCs and the SKBR3 cells. Usually, normal MSCs have a higher content in saturated fatty acids than the cancer cell line. A change in the saturated fatty acid ratio could be observed in SKBR3 cells, which have a higher content compared to the MSCs at both concentrations of 0.25 mM and 0.5 mM CuNPs. Unsaturated fatty acids were found to be more abundant in the SKBR3 cell line compared to the MSCs.

## 3. Discussion

This study evaluated the cytotoxic effects of CuNPs on normal MSCs and SKBR3 cancer cells, integrating real-time monitoring, apoptosis assay, oxidative stress analysis, gene expression, mitochondrial respiration and fatty acid profiling. The cellular uptake of CuNPs is correlated with the particle size, with the smaller size NPs (30–50 nm) showing better uptake [18]. Another important factor influencing the interaction between CuNPs and cells is the type and morphology of the target cells. The spherical or quasi-spherical CuNPs used in this study are more efficiently uptaken, as confirmed in other studies as well [18]. Also, their small size (37.5 nm), confirmed by Pricop et al. [8], provides a better rate of internalization [19] and, at the same time, a higher capacity to induce oxidative stress through increased surface activity and ion release, leading to enhanced activation of the antioxidant defense mechanisms [20].

Therefore, the CuNPs synthesized and characterized in the previous study were used to obtain preliminary results on their cytotoxicity on the SKBR3 cell line [8]. The present study confirms the dose-dependent cytotoxic effects and decrease in cell proliferation at high concentrations observed in the previous study, where the same CuNPs were used on the SKBR3 cell line [8]. The cytotoxic effects of the same synthesized CuNPs were evaluated by the same real-time xCELLigence method but at different concentrations and on two different cell models, MSCs and SKBR3. The current study demonstrated a low-dose stimulating response at 0.25 mM, where both MSCs and SKBR3 cells showed a transient increase in the cell index compared to the control. However, at higher concentrations, CuNPs elicited a significant dose-dependent decline in the cell index, with the SKBR3 cell line showing greater sensitivity. These findings indicate that tumor cells, despite their high proliferative potential, are more vulnerable to redox imbalances and nanoparticle-induced stress than primary mesenchymal stem cells.

CuNPs could induce both cellular necrosis and apoptosis, as demonstrated in previous research [21]. Therefore, Annexin/PI staining assay was used to evaluate the apoptotic process triggered by CuNPs. In MSCs a more gradual apoptotic response was observed, while the SKBR3 cells exhibited an increase in apoptosis and necrosis only at higher doses. The induction of oxidative stress and apoptosis following CuNP addition has also been observed in other types of cancer cells like the MCF7 breast cancer cell line and SW480 human colon cancer cell line, following a 24 h treatment [22,23].

Under oxidative stress, cells typically respond by upregulating their antioxidant defense systems to mitigate ROS-induced damage. However, when these defense mechanisms are overwhelmed, excessive ROS production can lead to protein oxidation, lipid peroxidation [24], DNA damage, mitochondrial dysfunction and, ultimately, apoptosis [25,26,27]. A more common toxic effect in CuNP-treated cells is ROS production, followed by apoptosis and/or necrosis [28]. Fluorescence microscopy revealed higher basal ROS levels in MSCs, which can be associated with their intrinsic biological properties. This may reflect the greater reliance of MSCs on moderate ROS levels in regulating stem cell signaling and in the maintenance of cellular homeostasis and pluripotent capacity, whereas SKBR3 cells exhibit lower basal mitochondrial ROS production, likely because they rely more on glycolytic metabolism, as is typical for many cancer cell types [29]. MSCs are primary cells with robust esterase function and physiological requirements for ROS, which appear to be a necessary condition for the initiation of stem cell proliferation after exiting the quiescent state [30]. In MSCs, higher basal esterase activity reflects an active metabolic state, supported by moderate ROS concentrations associated with their active proliferative and pluripotent status. This redox-regulated balance maintained by the antioxidant system promotes controlled cell renewal while preventing oxidative damage.

Cancer cells exposed to CuNPs have a higher capacity to produce ROS than normal cells because cancer cells have higher metabolic rates. CuNPs intensify the oxidative stress, increasing the ROS levels above the survival threshold and overwhelming the compensatory cellular antioxidant defense mechanisms. This makes them particularly efficient for selectively targeting cancer cells that are already under oxidative stress [31]. Consistent with this observation, the quantitative PCR results revealed that MSCs maintained relatively stable expression of antioxidant genes following CuNP exposure. The stability of antioxidant gene expression in MSCs suggests an efficient basal redox buffering system, enabling them to preserve homeostasis in response to CuNP-related oxidative stress. In contrast, SKBR3 cells displayed increased expression of antioxidant genes, particularly *CAT* and *SOD*, reflecting a compensatory mechanism to counteract elevated ROS levels, as also revealed by strong dose-dependent fluorescence observed after DCFH-DA staining. This strong transcriptional activation indicates that tumor cells are more prone to oxidative imbalance when exposed to CuNPs [14]. The differential antioxidant genes’ response in MSC and SKBR3 cells indicates that CuNP-induced oxidative stress surpasses the adaptive threshold of SKBR3 cells but nevertheless remains within the compensatory response range of the MSCs.

Mitochondrial respiration assessment revealed that CuNPs drastically impaired the respiratory capacity in both MSCs and SKBR3 cells. The SKBR3 cells were noticed to have a higher baseline respiratory capacity compared to MSCs, which is consistent with their elevated metabolic demands; however, CuNP exposure caused a dose-dependent reduction of routine respiration, OXPHOS and uncoupled capacity in both MSCs and SKBR3 cells. The progressive, dose-dependent decline in mitochondrial respiration suggests that CuNPs impair mitochondrial function primarily through oxidative stress and electron transport chain disruption, with SKBR3 cells showing greater sensitivity than MSCs due to their lower antioxidant capacity. These results point towards mitochondria as key targets, because CuNPs disrupt mitochondrial activity, leading to cellular damage and reducing energy metabolism [2].

Beyond mitochondrial dysfunction, CuNPs can also affect lipid metabolism. Previous studies have showed that excessive copper concentrations could induce mitochondrial dysfunction, promoting lipid deposition and lipogenesis [15]. The examination of the fatty acid profile by a sensitive analytical technique like gas chromatography/mass-spectrometry (GC-MS) can be a promising tool in cancer diagnosis and in the critical assessment of the NPs’ therapeutic potential [32]. High doses of CuNPs induced alterations in the cellular lipid profiles and the activation of inflammatory mechanisms [33]. The most prominent change of individual saturated fatty acids (SFAs) is C16:0, palmitic acid, which has the lowest level in SKBR3 cells, and it does not change in the CuNP-treated cells. Palmitic acid is a signaling molecule acting through the induction of post-translational modifications of proteins and as an on–off switch for protein activity. In cancer cells, palmitic acid enhances proliferation, metastasis and invasiveness by stabilizing oncogenic proteins [34]. In our study, palmitic acid was found to be at higher levels in MSCs compared to the SKBR3 cells. Elevation of SFA levels indicates a potential protective mechanism against oxidative stress, due to lower levels of the substrate for peroxidation, without being accompanied by a decrease in PUFAs. Due to their structure, polyunsaturated fatty acids (PUFAs) are molecules vulnerable to peroxidation, resulting in reactive aldehydes as end products [35]. Tumor cells can change their fatty acid content to modulate sensitivity to lipid peroxidation or as a response to the diseased state [36]. PUFAs, like linoleic acid, arachidonic acid and eicosapentaenoic acid, are substrates for prostaglandin production [37], being involved in inflammatory processes and oxidative stress; therefore, their higher content in the SKBR3 cells may affect critical intracellular signaling pathways [35]. PUFAs were found at higher concentrations in the control and the CuNP-treated SKBR3 cells compared to the MSCs, except for the MSCs treated with the 1 and 2 mM CuNP concentrations, where arachidonic and eicosapentaenoic acids were at higher levels. Oleic acid (C18:1) has different effects in various cancer cell types: in some, it induces apoptosis while in others stimulating cell growth [38]. In our study, oleic acid increased in SKBR3 cells compared to MSCs; this fact confirms the role of this fatty acid in promoting cell proliferation [39], but this effect was reversed in the 0.5 mM concentration treated cells, meaning that this concentration inhibited cancer cell proliferation. The same effect was observed in the elaidic acid behavior, with this being the isomer of oleic acid.

Our research pointed out that CuNPs induced cytotoxicity at higher concentrations (1–2 mM). The study suggested that higher CuNP concentrations caused apoptosis, especially in SKBR3 cells, which positively correlates with lower mitochondrial respiration. CuNPs interfered with the expression and activities of several antioxidant enzymes, including superoxide dismutase (*SOD*) and catalase (*CAT*), inducing oxidative stress and increasing ROS generation. CuNP-induced mitochondrial dysfunction and apoptosis is most likely due to the oxidative stress causing alterations in lipogenic metabolism. Herein, we showed that the oxidative stress triggered by the 1 mM CuNP concentration in the SKBR3 cells acted as a signaling mechanism, increasing lipogenesis via *PPARγ* (lipogenic gene).

The present study has both strengths and limitations. The strong points relate to the new data regarding the in vitro effects of CuNPs on a breast cancer cell line compared to normal MSCs. The cytotoxic effects were analyzed using a continuous screening method, through which results could be obtained in real time without affecting the mitochondrial activity. The following assays used to evaluate different metabolic pathways were general methods also used in preclinical and medical evaluations, such as flowcytometric assays (apoptosis measurement), microscopy (ROS generation assay), molecular biology (gene expression) and spectrometric analytical screening methods (GC-MS). FAME profiles evaluated through GC-MS could represent an innovative screening method for potential specific biomarkers and could lead to a specific type of treatment in cancer. The strategy of normal and cancer cell evaluations at the same time using the same methods provides new mechanistic insights into how CuNPs differentially modulate redox balance and metabolic responses according to cellular phenotype, thereby advancing the understanding of their potential biomedical use and limitations. The limitations of the study outline prospects for future experiments. A different form of CuNP synthesis could lead to more effective toxicity against cancer cells and fewer side effects on normal cells. The stability and hydrodynamic size of CuNPs in cell culture media were not characterized, which could influence the in vitro interpretation of their biological interactions and mechanistic behavior. Also, CuNPs should also be tested on other types of normal and cancerous cell lines. Therefore, a deeper investigation into the role of CuNP effects on lipids metabolism correlated with the fatty acid profiles is warranted. Oxidative stress is a complex process leading to the generation of various types of free radicals (e.g., oxygen, nitrogen species, etc.), and the markers of lipid peroxidation should also be extensively analyzed. Other detailed investigations should rely on measuring protein expression levels and performing observations at subcellular levels through electron scanning and transmission microscopy. All in vitro studies offer data for in vivo translation, which could offer a strong basis for clinical applications in human subjects.

## 4. Materials and Methods

### 4.1. CuNP Synthesis and Characterization

Copper nanoparticles (CuNPs) were synthesized via a chemical reduction method involving a two-step procedure, a technique adapted from our previous work [40]. In the first stage, trisodium citrate dihydrate (TSC) (ACS reagent, >99% purity) functioned simultaneously as a stabilizing and complexing agent. Subsequently, sodium borohydride (NaBH_4_) (Chimreactiv SRL, Neamt, Romania) was introduced to serve as the reducing agent, facilitating the reduction of Cu(II). The optimal synthesis parameters were established at a molar proportion of Cu(II):TSC:BH_4_^−^ = 1:1:0.2, pH = 5, with continuous stirring for 60 min at ambient temperature (25 °C). Under these conditions, uniform and well-dispersed CuNPs with an average size of approximately 37.5 nm were obtained. The synthesized CuNPs were examined using scanning electron microscopy (SEM) coupled with energy-dispersive X-ray spectroscopy (EDX) (Quanta FEG 250 Scanning, FEI Company, Hillsboro, OR, USA) to evaluate their morphology and elemental composition. To complement the experimental data, the Mie theory was applied to estimate the theoretical size of the copper nanoparticles, using, at first, a theoretical spectrum “C_ext_/C_sca_/C_abs_ vs. wavelength”, where C_ex_ was converted into the molar absorption coefficient ε, from the Lambert–Beer Law, using Equations (1) and (2) [40]:

(1) $$\varepsilon =N_{A}\cdot C_{ext}\cdot \ln10\cdot (10^{-3}\frac{L}{{cm}^{3}})\left[L/mol\times cm\right]$$

where N_A_ = 6.022 × 10^23^ (Avogadro’s constant) [L/mol], and C_ext_ = extinction cross-section [cm^2^]

(2) A=ε⋅1⋅c

and where A is the absorbance; ε is the molar absorption coefficient (M^−1^, cm^−1^); c is the concentration of the solution (M); and l is the path length (cm).

This approach allows for the calculation of light–particle interactions when the particle dimensions are comparable to the wavelength of incident light, providing valuable insights into their optical and structural behavior. The computations were conducted using MiePlot v4.6 (Philip Laven, Wokingham, UK), which contains reference datasets suitable for modeling spherical CuNPs.

### 4.2. Impedance-Based Cytotoxicity Assay

The impact of CuNPs was investigated on both normal MSCs and the SKBR3 breast cancer cell line. The MSCs were derived from dental pulp tissue and isolated in accordance with approved ethical protocols. The SKBR3 cell line was obtained from American Type Culture Collection (ATCC, HTB-30, Manassas, VA, USA). To monitor the influence of CuNPs on cellular proliferation, the real-time cell analysis system xCELLigence (Santa Clara, CA, USA) was employed. The system uses specially designed microtiter plates with interdigitated gold electrodes, which can detect changes in electrical impedances. The changes in electrical impedance are monitored and are used as an indirect sensor of cell viability and adherence in a non-invasive and label-free manner. To assess their viability in response to CuNPs, the cells were seeded at a density of 7 × 10^3^ cells per well and allowed to adhere overnight. The following day, CuNPs were added at 0.25 mM, 0.5 mM, 1 mM and 2 mM concentrations. Control groups consisted of untreated cells maintained in fresh cell culture medium. The cells were observed for 7 consecutive days, and the data were analyzed using RTCA software 1.2 version 1.2.1 (Santa Clara, CA, USA).

### 4.3. Assessment of Apoptosis via Annexin V FITC/PI Staining

To investigate the pro-apoptotic effects of CuNPs on normal MSCs and the SKBR3 cancer cell line, the Annexin V FITC/Propidium iodide (PI) staining method was used, followed by flowcytometric acquisition and analysis. The cells were seeded at a density of 5 × 10^5^ cells per well and allowed to adhere overnight. The following day, CuNPs were added at concentrations of 0.25 mM, 0.5 mM, 1 mM and 2 mM for 24 h in complete culture medium. The choice of exposure time interval was made to balance cytotoxicity assessment with preservation of viable cells for subsequent metabolic assessment. At this point, both MSCs and SKBR3 cells exhibit clear evidence of apoptosis, confirming the cytotoxic potential of CuNPs, yet a substantial fraction of cells remained viable, enabling subsequent evaluation of downstream metabolic parameters that we investigated in this study: mitochondrial respiration, changes in the fatty acid profiles and gene expression. Following treatment, cells were harvested, washed twice with phosphate-buffered saline (PBS) and resuspended in 1× Binding buffer. Staining was performed using Annexin V FITC and PI (5 µL each) according to manufacturer’s instructions for the FITC Annexin V Apoptosis Detection kit I (BD, Erembodegem, Belgium). After 15 min incubation, 400 µL of 1× Binding buffer was added, and the cells were analyzed by flow cytometry on a BD FACSAria III flow cytometer (Becton Dickinson Biosciences, Franklin Lakes, NJ, USA). Apoptotic and necrotic cells were subsequently quantified using a free online version of Floreada.io software 3/30/25 (https://floreada.io).

### 4.4. Reactive Oxygen Species Detection Assay

To assess the oxidative stress response triggered by CuNP exposure, intracellular reactive oxygen species (ROS) levels were quantified using the fluorescent probe 2,7 dichlorofluorescein diacetate (DCFH2-DA). Cells were seeded in IBIDI culture plates (Ibidi GmbH, Grafelfing, Germany) at a density of 2 × 10^4^ cells per well and allowed to adhere overnight. The next day, CuNPs were added at specific concentrations (0.25 mM, 0.5 mM, 1 mM and 2 mM), and the cells were treated for 24 h. Untreated cells cultured in fresh medium served as controls. Following treatment, the cells were washed with Dulbecco’s Modified Eagle Medium (DMEM) (Sigma Aldrich, St. Louis, MO, USA), stained with 10 µM DCFH2-DA and returned to the incubator for 30 min, at 37 °C. Following incubations, cells were washed in PBS and observed under a Zeiss Axio Observer.D1 phase contrast microscope with fluorescence (Zeiss, Oberkochen, Germany).

### 4.5. Oxidative Stress-Related Gene Expression

In order to evaluate oxidative stress gene expression, the cells were seeded at a density of 5 × 10^5^ cells per well and allowed to adhere overnight. The following day, CuNPs were added at concentrations of 0.25 mM, 0.5 mM, 1 mM and 2 mM for 24 h in complete culture medium. Total RNA was extracted using Trizol reagent (Invitrogen, Carlsbad, CA, USA), following the manufacturer’s protocol. Complementary cDNA was generated from 1 µg of total RNA using a RevertAid First Strand cDNA Synthesis kit (Thermo Scientific, EU, Vilnius, Lithuania). The resulting cDNA was subsequently used as a template for quantitative real-time PCR, performed with KiCqStart SYBR green qPCR Ready Mix (Sigma, St Louis, MO, USA) on a Light Cycler 480 system (Roche, Basel, Switzerland). Primer sets were specifically designed to amplify genes involved in oxidative stress regulation pathway and included *PPARγ*, superoxide dismutase (*SOD*) and catalase (*CAT*). Primer sequences are listed in Supplementary Table S1.

### 4.6. Assessment of Mitochondrial Respiration

To evaluate mitochondrial function, the cells were seeded at a density of 5 × 10^5^ cells per well and allowed to adhere overnight. The following day, CuNPs were added at the same concentrations of 0.25 mM, 0.5 mM, 1 mM and 2 mM for 24 h in complete culture medium. Following treatment, cells were harvested by trypsinization and resuspended in complete cell culture medium. To evaluate the effects of CuNPs on mitochondrial function, oxygen consumption rates were measured using the high-resolution Oxyograf 2k respirometry system (Oroboros Instrument, Innsbruck, Austria). Baseline respiration was first recorded under physiological conditions. To assess proton-leak and non-ATP respiration, 2.5 µM oligomycin was added to inhibit ATP synthase. The maximal electron transport system capacity was determined by several titration steps of the uncoupler FCCP. Rotenone and antimycin A were subsequentially introduced to inhibit complex I and III of the respiratory chain, enabling the measurements of the residual oxygen consumption. All reagents used were acquired from Sigma-Aldrich (St. Louis, MO, USA).

### 4.7. Fatty Acid Methyl Ester Extraction and GC-MS Content Analysis in Cells

All solvents and reagents used for extraction, derivatization and GC-MS were chromatography grade, obtained from Sigma-Aldrich (St. Louis, MO, USA). The fatty acid (FA) content of cells (5 × 10^5^ cells per experiment, *n* = 3) was evaluated. Cells were cultivated and collected for extraction but, before, were washed three times with ice-cold PBS, being centrifuged at 1500 rpm for 10 min each time. The obtained pellet of cells was used for FA extraction in two steps. In the first step, the pellet was extracted using the Folch et al. (1957) method [17]. Therefore, 400 µL of chloroform/methanol (2:1) solution was added to the pellet, and then vortexed and centrifuged at 4000 rpm for 5 min. The chloroform layer was transferred and mixed with 200 µL of water and 100 µL of chloroform. The obtained mixture was vortexed and centrifuged at 4000 rpm for 10 min. Again, the chloroform layer was collected and evaporated to dryness under a 1 L/min constant flow of inert gas (nitrogen) [17]. The second step consists in the saponification and extraction processes. The dried residue was mixed with 0.45 M sodium hydroxide methanolic solution and heated at 65 °C for 1 h and then neutralized with 0.45 M HCl. The mixture was extracted two times with 2 mL of 2% acetic acid in hexane. The hexane layer was collected and dried under nitrogen gas. The third step includes the derivatization of FAs into fatty acid methyl esters (FAMEs). The resulting residue was mixed with 100 µL of acetyl chloride in 5 mL of methanol under continuous agitation for 45 min. The reaction was stopped with 3 mL of 0.25 M potassium carbonate, and the resulting methyl esters were extracted in 1 mL of hexane under continuous agitation for 1 h. The organic hexane layer was collected and dried under nitrogen gas. The dried residue was resuspended in 100 µL of hexane, and 1 µL of sample was injected into the GC-MS system. Gas chromatography analyses were conducted on an Agilent 6890 gas chromatograph coupled with a 5973 MSD quadrupole mass spectrometer (Agilent Technologies, Santa Clara, CA, USA). The separation of compounds was performed on a DB-WAX capillary column (30 m × 0.25 mm × 0.25 µm) with a flow rate of 1 mL/min of helium. The oven temperature started from 50 °C, increasing to 250 °C with a constant rate of 6 °C/min. Samples were injected into an inlet heated to 230 °C in splitless mode. The mass spectrometer source was heated at 150 °C and was set to analyze compounds after a solvent delay of 4 min with a standard ionization energy of 70 eV. Compounds were scanned in full-scan mode, with masses ranging from *m*/*z* 50 to 600 Da. Compounds were identified by comparing the experimental spectra to the NIST11 mass spectral library (>95% of similarity) using ChemStation software, version B.01.00 (Agilent Technologies, Palo Alto, CA, USA). The results were presented as the mean of abundances calculated from three independent experiments. Then, all values were normalized to the number of cells (5 × 10^5^ cells/sample), and a percentage area was calculated. The percentage area for each sample was calculated by dividing it with the total area (obtained by summing all FAMEs’ areas) and multiplying by 100. To ensure accuracy and eliminate contamination risks, solvent blanks and a FAME standard were analyzed under identical GC-MS conditions.

### 4.8. Statistical Analysis

All statistical analyses and graphical summaries of the data were performed using the Data Analysis Tool of Microsoft^®^ Excel^®^ for Microsoft 365 MSO (Version 2502 Build 16.0.18526.20168), using 32-bit (Microsoft Corporation, Redmond, WA, USA) package. Differences between experimental groups were assessed using one-way ANOVA followed by a pairwise post hoc *t*-test to determine specific group differences. Statistical significance was established at a threshold of *p* < 0.05; data are presented from three independent experiments as the mean ± standard deviation (SD).

## 5. Conclusions

CuNPs synthesized through a chemical method seem to be an effective therapeutic option for certain cancers, as demonstrated in our work through cytotoxicity, ROS gene expression, mitochondrial respiration and lipid metabolism assays. However, the chemical formulation and cytotoxicity potential of CuNPs on both normal and cancer cells must be very carefully evaluated, since prolonged or excessive exposure to certain formulations of CuNPs can be accompanied by the generation of oxygen reactive species and release of copper ions, factors that represent major challenges limiting their biomedical applications. In our study, at the functional level, CuNP exposure induced a marked loss of cell viability in a dose-dependent manner, with the SKBR3 breast cancer cell line being more vulnerable than normal MSCs. Apoptosis was identified as the primary mode of cell death after 24 h exposure time, occurring in a dose-dependent manner and accompanied by a smaller proportion of necrotic cells. Mitochondrial respiration assays revealed profound impairment in both cell types, underlining mitochondrial dysfunction as a central mechanism of CuNP-induced cytotoxicity. In parallel, lipid profiling indicated clear differences between SKBR3 breast cancer cells and normal MSCs when exposed to CuNPs. Also, the GC-MS analyses indicate that unsaturated fatty acids may be overproduced in CuNP-treated cells, with an increased tendency in breast cancer cells, serving as potential precursors for the production of cancer cell-specific PUFAs. MSCs have a higher percentage of saturated fatty acids than the SKBR3 cells, whereas SKBR3 cells showed an altered lipid composition, reflecting differences in lipid homeostasis and increased sensitivity to oxidative stress. These results support the concept that cells with different malignancy profiles show distinct steady-state lipid content and sensitivity to oxidative stress. Taken together, the interaction between mitochondrial damage, oxidative imbalance and lipid remodeling highlights the complex, multi-layered nature of CuNP-induced cytotoxicity in cancer cells versus normal stem cells, effects that could be exploited in future personalized approaches to cancer therapy.

## Data Availability

Data are contained within the article and Supplementary Materials.

## Supplementary Materials

The following supporting information can be downloaded at: https://www.mdpi.com/article/10.3390/ijms262110716/s1.

## Abbreviations

The following abbreviations are used in this manuscript:

NPs	Nanoparticles
MSCs	Mesenchymal Stem Cells
GC-MS	Gas Chromatography–Mass Spectrometry
qPCR	Quantitative Polymerase Chain Reaction
PPARγ	Peroxisome Proliferator-Activated Receptor Gamma
CAT	Catalase
ROS	Reactive Oxygen Species
DNA	Deoxyribonucleic Acid
PBS	Phosphate-Buffered Saline
MEM	Minimum Essential Medium
FCS	Fetal Calf Serum
ATP	Adenosine Triphosphate
cDNA	Complementary DNA
RNA	Ribonucleic Acid
OXPHOS	Oxidative Phosphorylation
SOD	Superoxide Dismutase
FAMEs	Fatty Acid Methyl Esters
FAs	Fatty Acids
PPARs	Peroxisome Proliferator-Activated Receptors
