# Supplementary material for: Cellular Metabolic Responses to Copper Nanoparticles: Comparison Between Normal and Breast Cancer Cells
Source: Int J Mol Sci. 2025 Nov 4;26(21):10716. doi: 10.3390/ijms262110716 (PMC12609715; doi:10.3390/ijms262110716)
Supplement: Supplementary file 1 [file ijms-26-10716-s001.zip › ijms-3943616-supplementary.pdf]

Supplementary materials

Cellular Metabolic Responses to Copper Nanoparticles: Comparison Between Normal and Breast Cancer Cells

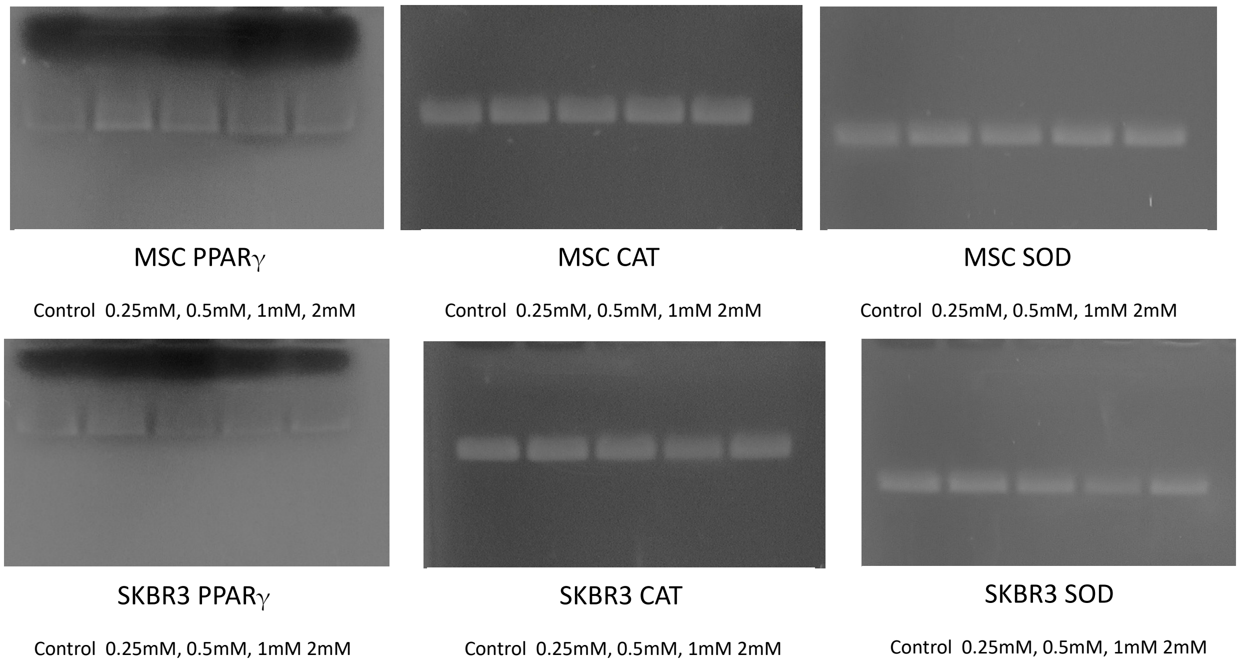

Figure S1. Representative agarose gel electrophoresis analysis of qPCR products targeting oxidative stress-related genes (PPARγ, SOD, CAT)

Table S1. List of primer sequence for quantitative PCR (q-PCR) analysis

Gene	Primer sequence
PPARγ	AAGACCACTCCCACTCCTTTG
	GTCAGCGGACTCTGGATTCA
SOD	GGTGGGCCAAAGGATGAAGAG
	CCACAAGCCAAACGACTTCC

CAT	TGGAGCTGGTAACCCATGAGG
	CCTTTGCCTTGGAGTATTTGGTA

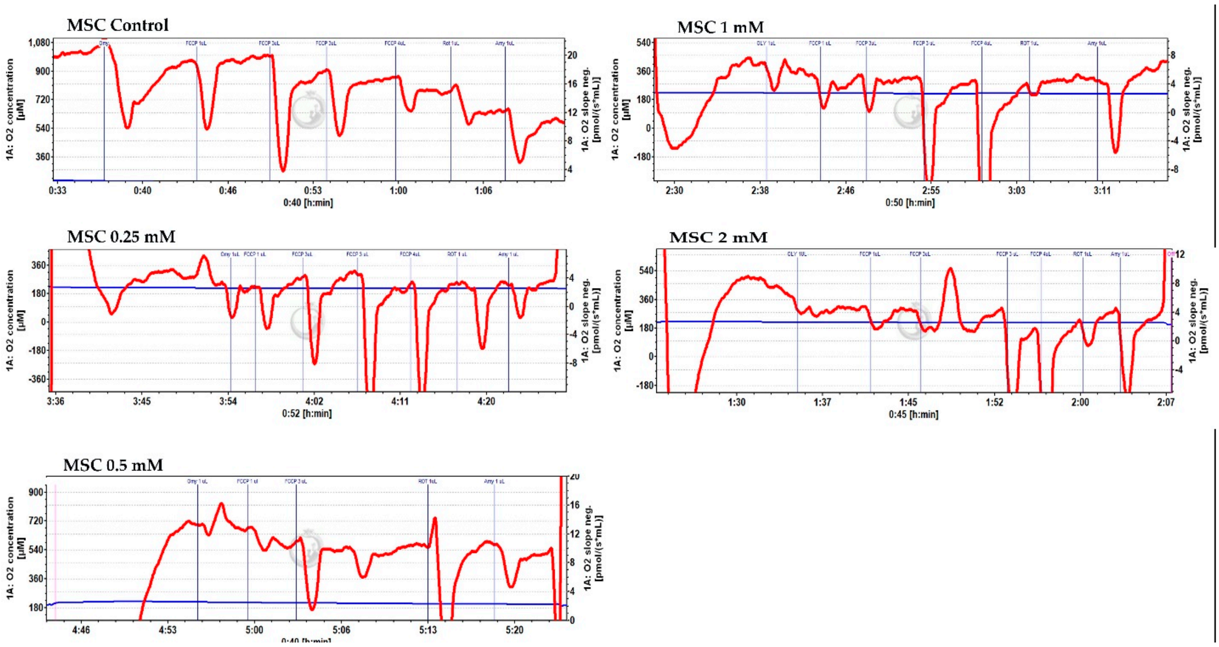

Figure S2. Representative Oroboros oxygraphs showing oxygen consumption profiles of MSC cells treated with different concentrations of CuNPs. Basal respiration was recorded first, followed by oligomycin-insensitive leak respiration. FCCP addition revealed maximal respiratory capacity, while rotenone and antimycin A fully suppressed mitochondrial activity to define non-mitochondrial oxygen consumption.

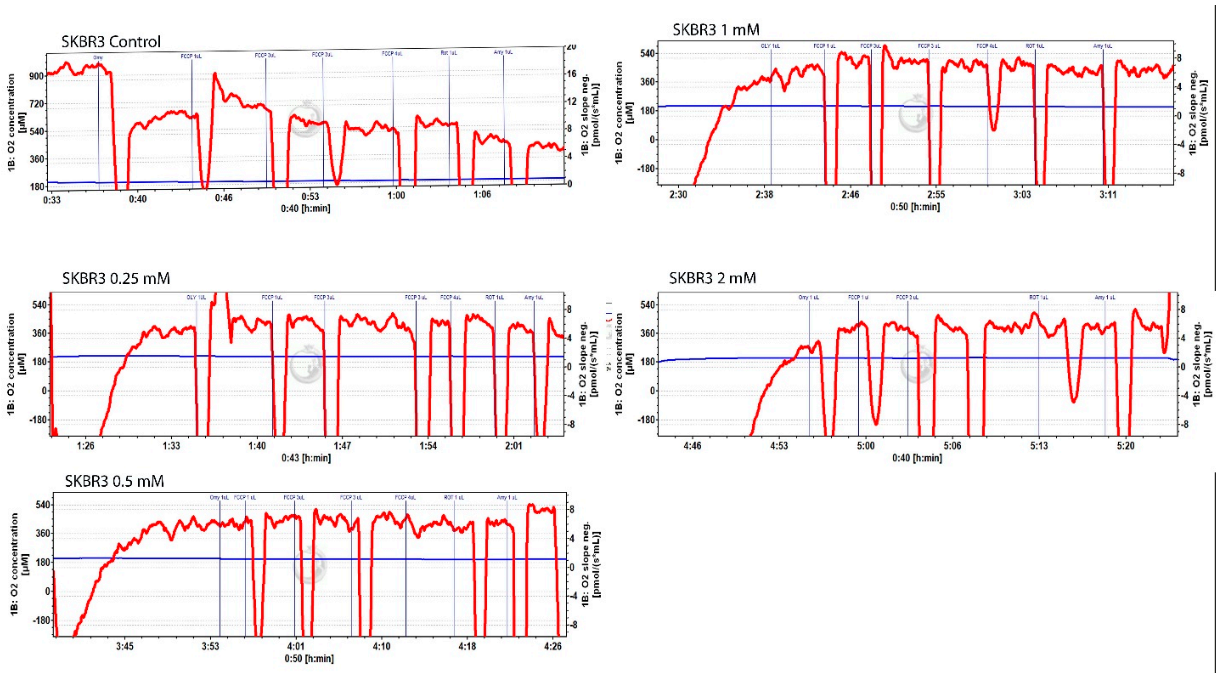

Figure S3. Representative Oroboros oxygraphs illustrating oxygen consumption profiles of SKBR3 cells exposed to different concentrations of CuNPs. The traces show basal respiration, oligomycin-insensitive leak respiration, maximal respiratory capacity after FCCP addition, and non-mitochondrial oxygen consumption determined following rotenone and antimycin A treatment.

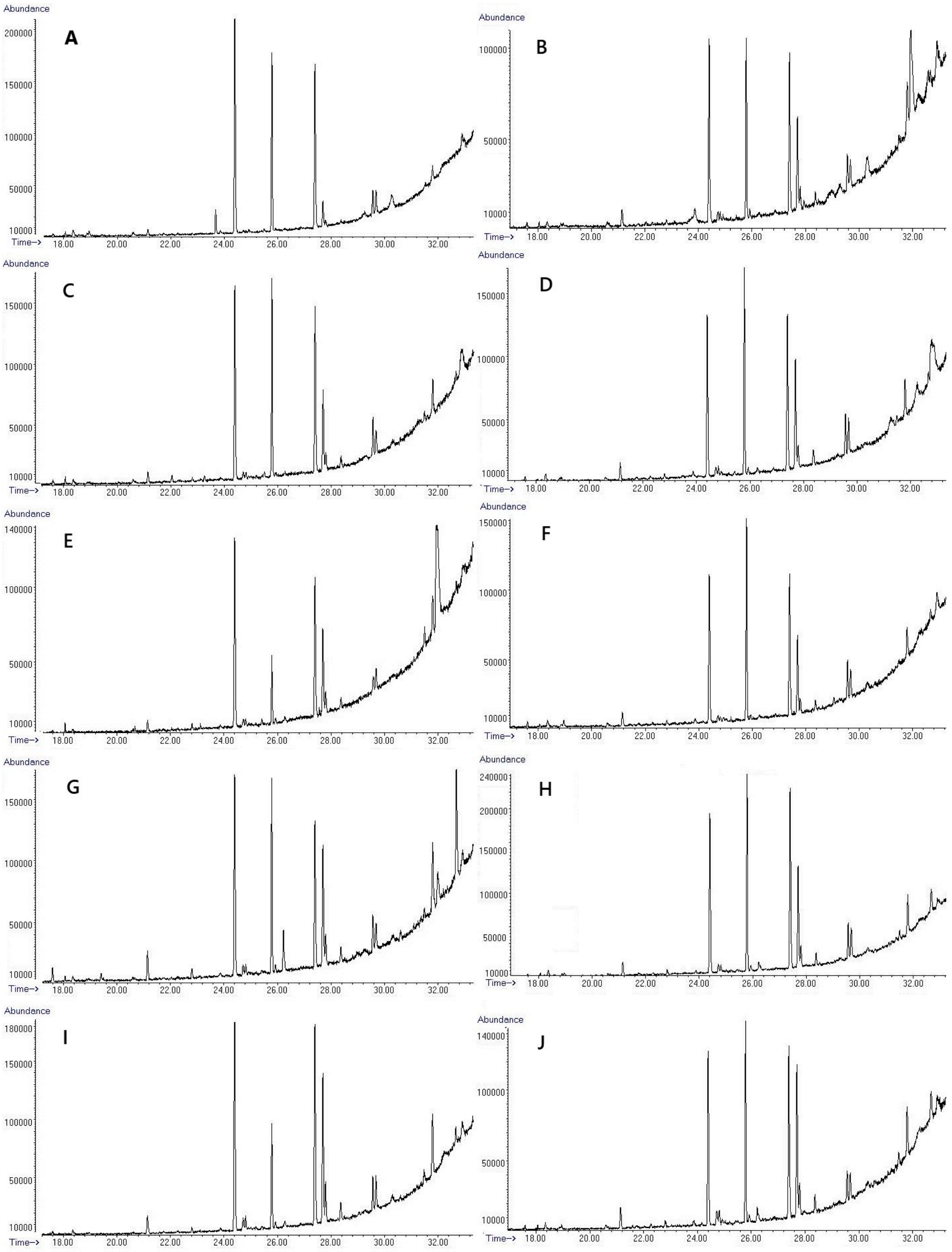

Figure S4. Representative raw TIC (total ion current) chromatograms for MSC and SKBR3 cell lines: A. MSC cells control; B. SKBR3 cell line control; C. MSC cells treated with 2 mM CuNPs; D. SKBR3 cell line treated with 2 mM CuNPs; E. MSC cells treated with 1 mM CuNPs; F. SKBR3 cell line treated with 1 mM CuNPs; G. MSC cells treated with 0.5 mM CuNPs; H. SKBR3 cell line treated with 0.5 mM CuNPs; I. MSC cells treated with 0.25 mM CuNPs; J. SKBR3 cell line treated with 0.25 mM CuNPs. FAMEs data were normalized to $5*10^5$ cells/sample (n=3; n, number of samples).
